# Flying in the Face of the Evidence; Low Aspirin Use in the US Outpatient Setting

**DOI:** 10.1371/journal.pmed.0020416

**Published:** 2005-11-15

There are many instances in medical practice of an intervention becoming adopted despite evidence of it not being of the highest quality. The converse is also true; despite good evidence for the effectiveness of a treatment, it may not be implemented for a variety of different reasons. Reasons may be social or financial or due to a lack of knowledge of the benefits of the intervention.

In this issue of *PLoS Medicine*, Randall Stafford and colleagues investigate the use of one such underused intervention, aspirin, in the United States. Many studies have shown that aspirin is beneficial as both primary and secondary prevention of cardiovascular disease in a wide range of patients who do not otherwise have contraindications such as increased bleeding risk. For example, the American College of Chest Physicians has issued a grade 1A recommendation (such recommendations are “strong and indicate that the benefits … outweigh risks, burden, and costs”) that aspirin is used for secondary prevention of acute coronary events. The American Diabetes Association recommends aspirin to all people with diabetes over 40, or younger if they have additional cardiovascular risk factors.

Stafford and colleagues used the 1993–2003 US National Ambulatory Medical Care Survey (NAMCS) and National Hospital Ambulatory Medical Care Survey (NHAMCS) to estimate aspirin use by cardiovascular risk. These validated surveys are comprehensive collections of data on prescribing; NAMCS captures health-care services provided by private office-based physicians, whereas NHAMCS captures services offered at hospital outpatient departments. Their data are representative of care provided in a huge number of outpatient visits—around 750 million in 2003.

What the authors found was that although aspirin use increased steadily over the time of the survey, even at its highest—in 2003—it was prescribed at only 32.8 % of high-risk visits; for low-risk visits, the rate was just 1%–3%. They also compared prescribing of aspirin and statins, and found that the rates of prescribing of statins overtook that of aspirin in 1997–1998, and rose steadily thereafter. There were also substantial differences in the age of patients prescribed aspirin, with lower usage found in those below 45 than those 45 or above, and lower usage in women. Also, there was lower prescribing by noncardiologists than cardiologists, in private practices versus hospital outpatient departments, and at return visits versus first-time visits. In people with diabetes who were at intermediate risk, aspirin use was only 11.7% by 2003.

So despite all the recommendations and evidence for the use of aspirin, rates of prescribing are low, even for conditions where clear guidelines exist. As the authors say, “gaps observed with secondary prevention are particularly concerning, given the existence of conclusive clinical evidence and unequivocal practice guidelines.” One particular cause of this lack of adherence to guidelines may be specific to the US—the less restrictive regulations on newer drug advertising of drugs such as statins, particularly widespread consumer advertising. Hence, despite good evidence and the cost effectiveness of aspirin, statins are increasingly preferred over aspirin

As the authors conclude, “marked changes in clinical practice are unlikely to occur unless more aggressive, innovative means are implemented to enhance health-care provider and patient adherence to consensus guidelines.”

